# Stand-Alone Lateral Interbody Fusion for the Treatment of Low-Grade Degenerative Spondylolisthesis

**DOI:** 10.1100/2012/456346

**Published:** 2012-04-01

**Authors:** Luis Marchi, Nitamar Abdala, Leonardo Oliveira, Rodrigo Amaral, Etevaldo Coutinho, Luiz Pimenta

**Affiliations:** ^1^Department of Minimally Invasive Surgery, Instituto de Patologia da Coluna, São Paulo, SP 04101-000, Brazil; ^2^Department of Imaging Diagnosis, Universidade Federal de São Paulo, São Paulo, SP 04024-002, Brazil; ^3^Department of Neurosurgery UCSD San Diego, San Diego, CA 92103, USA

## Abstract

The purpose of this paper was to investigate the stand-alone lateral interbody fusion as a minimally invasive option for the treatment of low-grade degenerative spondylolisthesis with a minimum 24-month followup. Prospective nonrandomized observational single-center study. 52 consecutive patients (67.6 ± 10 y/o; 73.1% female; 27.4 ± 3.4 BMI) with single-level grade I/II single-level degenerative spondylolisthesis without significant spine instability were included. Fusion procedures were performed as retroperitoneal lateral transpsoas interbody fusions without screw supplementation. The procedures were performed in average 73.2 minutes and with less than 50cc blood loss. VAS and Oswestry scores showed lasting improvements in clinical outcomes (60% and 54.5% change, resp.). The vertebral slippage was reduced in 90.4% of cases from mean values of 15.1% preoperatively to 7.4% at 6-week followup (*P* < 0.001) and was maintained through 24 months (7.1%, *P* < 0.001). Segmental lordosis (*P* < 0.001) and disc height (*P* < 0.001) were improved in postop evaluations. Cage subsidence occurred in 9/52 cases (17%) and 7/52 cases (13%) spine levels needed revision surgery. At the 24-month evaluation, solid fusion was observed in 86.5% of the levels treated. The minimally invasive lateral approach has been shown to be a safe and reproducible technique to treat low-grade degenerative spondylolisthesis.

## 1. Introduction

Degenerative disc disease (DDD) in the lumbar spine can be associated with displacement of the superior vertebral body. Degenerative spondylolisthesis with concomitant spinal stenosis is among the most frequent conditions in the aging adult spine.

Treatment options for symptomatic spondylolisthesis continue to be discussed among spine professionals, but recent studies have shown that surgical procedures provided better improvement in pain and function compared to usual nonoperative care [1, 2]. The various surgical options have been studied to evaluate safety and optimal radiological and clinical results.

Posterior decompression has been thought to be a necessary component in treating spondylolisthesis with leg pain [3]. However, with posterior boney resection and wide muscle dissection this procedure can lead to spinal instability and deformity, [4]. Spinal fusion with decompression has been shown to result in better clinical outcomes [5, 6].

Surgical options, however, should seek to minimize collateral muscle/bone damage while achieving excellent clinical results, with minimal risk and complication rates. As a minimally invasive option, the lateral approach to interbody fusion avoids posterior-approach- and direct anterior-approach-related complications, achieves spinal stabilization and provides indirect decompression [7–12]. Additionally, the lateral approach preserves the inherent biomechanical integrity of the motion segment through maintenance of all the ligamentous structures, including the anterior longitudinal ligament (ALL) [7, 13, 14], which is considered to be one of the major stabilizing components of the lumbar spine [15].

The present study reports 24-month clinical and radiological results following stand-alone lateral interbody fusion for the treatment of single-level degenerative low-grade spondylolisthesis.

## 2. Materials and Methods

A prospective observational study of single-level L1-L5 degenerative low-grade degenerative spondylolisthesis following stand-alone lateral interbody fusion was conducted at a single institution under proper Ethical Committee's approval.

The inclusion criteria were single-level adult degenerative low-grade spondylolisthesis (Meyerding grades I-II) between L1 and L5, visual analog scale (VAS) for back pain ≥40 mm; Oswestry disability index (ODI) ≥ 50 points and radicular and/or axial lumbar pain. Exclusion criteria included prior fusion or arthroplasty surgery at the operative level, unstable and/or hypermobile levels (>3 mm translation, >11° rotation different from adjacent level) observed in dynamic X-ray images, trauma, tumor, or infection, and progressive neuromuscular disease.

Fifty-two patients fulfilled the study criteria. All patients were followed for a minimum of 24 months. Demographic and clinical data are shown on Table 1. The lumbar fusion procedure was performed through a minimally disruptive lateral, retroperitoneal, and transpsoas approach to the spine, performed in the standard fashion reported in previous studies [7–9]. Interbody spacers were large polyetheretherketone (PEEK) cages (NuVasive Inc., San Diego, CA, USA) with 18 or 22 mm wide in anteroposterior diameter and 45–55 mm wide in laterolateral diameter to lay on both lateral apophyseal ring. Cages were packed with calcium phosphate bone graft.

Clinical data was assessed through physical examination and patient-reported questionnaires (VAS and ODI) at every follow-up visit: preoperative, immediately postoperative, at 1 and 6 weeks, and 3, 6, 12, and 24 months. Digital X-ray, computed tomography (CT), and magnetic resonance (MRI) images were analyzed. Radiological parameters were reviewed by two observers other than the operating spine surgeons and a radiologist.

In lateral X-ray images, the following parameters were recorded: global lumbar lordosis from L1 to the sacrum segmental lordosis—the angle subtended by the superior endplate of the superior vertebral body and the inferior endplate of inferior vertebral body of the index level, mean disc height (average between anterior and posterior maximum disc heights), and relative amount of olisthesis expressed in percentage values of vertebral displacement. CT and X-ray images were reviewed to assess fusion. Fusion was defined as bridging bone connecting the adjacent vertebral bodies either through the implants or around the implants, less than 5° of angular motion, less than or equal to 3 mm of translation, and an absence of radiolucent lines around more than 50% of either of the implant surfaces. MRI studies were used for diagnosis and during followup as needed.

Subsidence was described as the radiologically observed loss of disc space height between the intervertebral cage and vertebral endplate from immediately postoperative to each of the follow-up time points. Subsidence was classified using an increasing severity scale: grade 0 = 0 to 24%, grade I = 25 to 49%, grade II = 50 to 74%, and grade III = 75% to total collapse of the level.

Statistical analyses were performed using SPSS software (SPSS, Version 10, SPSS, Chicago, Ill, USA). Student *t* test, *Z*-test for two proportions, test for equality of two or several population proportions, ANOVA and Pearson's correlation test were used for comparison between variables where appropriate, with a significance level of 0.05.

## 3. Results

### 3.1. Surgical and Clinical Results

Mean surgical duration was 73.2 ± 31.4 minutes (mean ± standard deviation). Blood loss averaged less than 50 mL. There were no intraoperative complications. In postoperative neurological examinations, 10 patients (19.2%) presented with psoas weakness, and five patients (9.6%) had anterior thigh numbness, both conditions resolving within 6 weeks in all cases.

**Figure 1:**
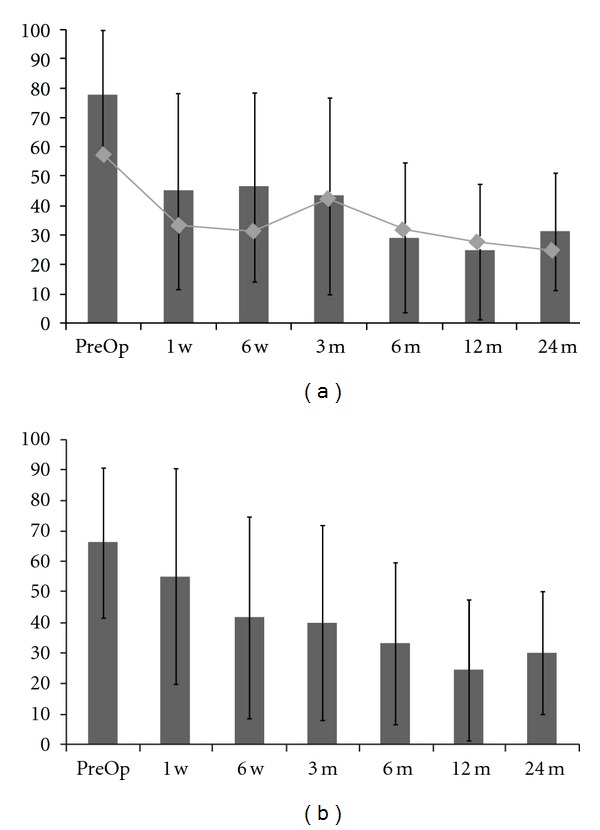
Clinical outcomes. (a) VAS back (columns) and legs (lines and dots) scores, all postoperative results are statistically significant compared to baseline (*P* < 0.05). (b) ODI scores, results are statistically significant (*P* < 0.05).

Clinical scores in patient-reported questionnaires (VAS back, VAS legs and ODI) showed fast and lasting pain relief and improvement in daily activities (Figure 1). Mean VAS back scores decreased from 78 to 45 mm at 1-week visit (*P* = 0.037) and 31 at final followup (*P* < 0.001). For leg pain assessment, mean VAS scores decreased from 54 to 31 mm at the 1-week visit (*P* = 0.001) and 23 at final followup (*P* = 0.007). Mean ODI scores were significantly improved at all postoperative visits compared with preoperative baseline and decreased from 66% to 30% along the study (*P* = 0.001).

### 3.2. Radiological Results

The analyzed radiological parameters are shown in Table 2. Mean preoperative vertebral slippage was 15.1% (min 6%, max 32%). Significant olisthesis reduction was shown at the 6-month visit (*P* < 0.001), which was maintained up to the last followup (*P* < 0.001). Also, disc space height was increased and maintained after intervertebral grafting (*P* < 0.001). Segmental lordosis at the index level was increased an average of 6.0° at 24 months (*P* < 0.001). Although global lordosis was statistically improved at the 6-week visit (42.8 to 48.5; *P* < 0.01), at the final followup, no difference was found for this parameter (42.8 to 46.5; *P* = 0.23). Fusion was observed in 86.6% of the levels treated. Seven levels were deemed to have incomplete bone ingrowth at 24-months on CT images; however, neither pseudoarthrosis nor movement at the index level were observed in those cases. None required revision due to pseudoarthrosis.

**Figure 2:**
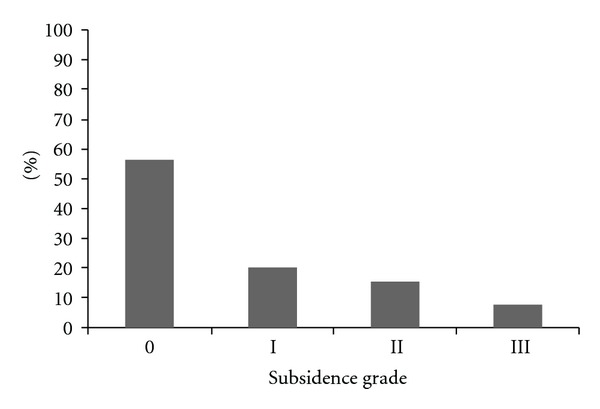
Subsidence occurrence at 12-month radiological assessment. Occurrence by grade: grade 0 : 55.8%, grade I : 26.9%, grade II : 11.5%, and grade III : 5.8%.

**Figure 3:**
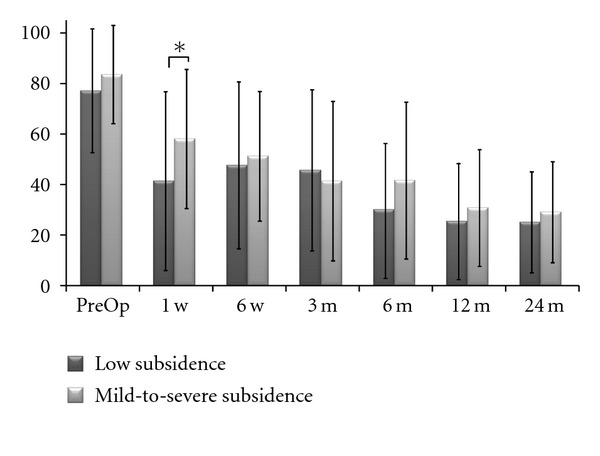
Clinical outcomes in different subsidence grade groups. VAS back in low-subsidence group (0 or I) and VAS back in mild-to-severe-subsidence group (II or III). *P = 0.045.

Cage subsidence was monitored postoperatively and expressed in grades of severity (Figure 2). 12-month and 24-month analyses revealed exactly the same results. Statistical analysis showed that this radiological phenomena was a low-grade occurrence (43 cases grade 0 or I in total 52 cases, 82.7%). Subsidence grade II and III occurred in 9 cases (17.3%). When patient data (demographic, clinical, and surgical) were evaluated with the subsidence subgroups (low or high grade), it was possible to identify some risk factors for developing significant subsidence (Table 3). Elderly and female patients tended to develop more severe subsidence (*P* = 0.019 and *P* = 0.041, resp.). Also, L4-5 seemed to be more susceptible to this event than upper levels, as there were more L4-5 cases in the grade II/III than in the grade 0/I subgroup (*P* = 0.038). Clinical manifestation of subsidence was evaluated through VAS back scores (Figure 3). Two groups were analyzed: (1) grade 0/I subsidence cases and (2) grade II/III cases. At the 1-week postoperative visit, back pain was increased in patients in the second group; this increase was not seen at later visits as the pain improved. Linear regression showed that the occurrence of subsidence had no correlation with either the amount of pre-op olisthesis (*r* ^2^ <0.001, *P* = 0.98) nor with slippage reduction (*r* ^2^ <0.001, *P* = 0.97).

### 3.3. Revision

Revision surgery was necessary for seven levels (13.5% of cases). Five revision cases had experienced high-grade subsidence with instability/restenosis. Other revisions were cases in which indirect decompression was not achieved. Revision surgeries were carried out minimally invasively to perform direct decompression and to add pedicle screws, to treat central/lateral stenosis, and to guarantee a rigid construction. No complications during revision surgeries were observed.

### 3.4. Case Examples

**Figure 4:**
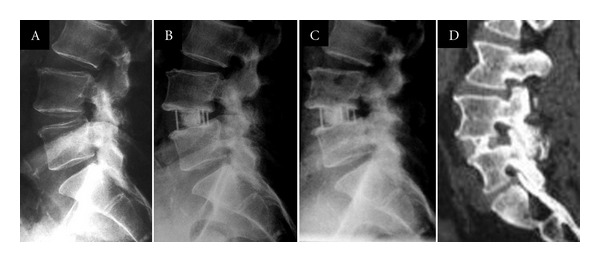
Case example #1. (a) Preoperative X-ray (b) 3-month X-ray (c) 12-month X-ray (d) 12-month computed tomography sagittal reconstruction.

A case example no. 1 is shown in Figure 4. Fifty-four-year-old, male, with significant leg and axial back pain, did not experience clinical improvement with conservative care. Neurogenic claudication was diagnosed with central and lateral stenosis at L4-5, which presented grade I degenerative spondylolisthesis. Stand-alone lateral interbody fusion was performed at L4-5. Postoperative exams revealed lasting disc height gain and olisthesis correction and at 24-month followup, bone fusion.

**Figure 5:**
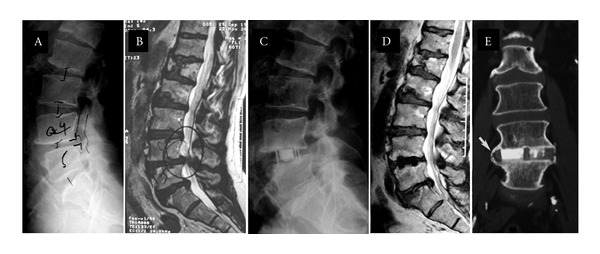
Case example #2. (a) Preoperative X-ray (b) Preoperative MRI sagittal reconstruction (c) 12-month X-ray (d) 24-month MRI sagittal reconstruction (e) 24-month computed tomography sagittal reconstruction. Arrow shows sentinel sign around cage.

In case example no. 2, a seventy-three-year-old male with central and lateral stenosis and neurogenic claudication was treated with lateral interbody fusion (Figure 5). Exams showed disc space gain, significant indirect decompression, slight slippage reduction, and bone fusion at last followup.

## 4. Discussion 

There is much discussion on spondylolisthesis management. Typical nonoperative care is based on nonsteroidal anti-inflammatories, physical therapy, local injections, weight loss, and exercise. But surgical intervention appears to be more effective in the treatment of degenerative spondylolisthesis and associated spinal stenosis, as reported by the randomized multicenter studies carried out by the Spine Patient Outcomes Research Trial (SPORT) [1, 2]. 

The anterior column transmits approximately 80% of the compressive load [16] and the facet joints play the major role in resisting torsional and shear loads [17]. Other biomechanical data show that the disc and ALL are stronger and stiffer in shear than the facet joints [18] and shear restraint depends directly on the tensile strain of the ligaments [19]. With disc degeneration, the tension in the ALL and inner annulus reduce, permitting a much larger shearing motion and forward displacement [20]. As opposed to spondylolytic spondylolisthesis, in degenerative spondylolisthesis the posterior neural arch and ligamentous complex tend to be intact. The insertion of an interbody space restores the tensile strain, augments the disc height, and corrects the anterior column alignment by indirect reduction of the subluxation [20, 21], thereby improving radiological and clinical parameters [21], as solid interbody fusion treats spine instability symptoms [22] and leads to better clinical outcomes compared with decompression alone [5, 22]. However, some studies suggest that aggressive fusion techniques do not necessarily guarantee good outcomes [1, 2]. Minimally invasive procedures may be sufficient to achieve these goals. 

Reconstruction of the anterior column can be performed via the direct anterior approach (transperitoneal or retroperitoneal), lateral approach (retroperitoneal), or via posterior techniques. 

Satisfactory anterolisthesis reduction, disc height restoration, and increased neuroforaminal height have been shown following TLIF procedures [22–24]. In addition to dissection and retraction of paraspinal musculature, posterior approaches inherently add some neurological risks to the procedures, such as epidural scarring, root damage and pseudoarthrosis [2, 25] although neuromonitoring during lumbar surgeries may help diminish the incidence of lumbar and sacral nerve root deficits [26]. Moreover, resection of the bony elements and ligaments of the posterior and middle column may cause spine destabilization [15] and consequent instrumentation failure, the most common complications on spondylolisthesis procedures [25]. 

Following the direct anterior approach, important spine stabilizers from the abdominal musculature become inactive, and other operative risks have to be considered, such as bowel perforation, incisional hernia, retrograde ejaculation, and vascular complications [27]. Moreover, the anterior wall of the spine (ALL and anterior annulus) which is responsible for spine stabilization on horizontal translation [15] is resected during a direct anterior interbody procedure. In addition to enabling slippage reduction by ligamentotaxis, interbody fusion with the lateral interbody construct preserves posterior and anterior elements providing the largest stand-alone reduction in range of motion compared with literature-reported ALIF and TLIF constructs [28]. 

The ability to reconstruct the anterior column after disc degeneration and slip is important in accomplishing the primary goals for surgery in patients with spondylolisthesis: (1) lasting segmental reduction and stabilization, (2) restoration of disc and neuroforaminal height, (3) correction of lumbar sagittal alignment, and (4) solid fusion. 

Slip reduction aims to reconstitute physiological spinal load-bearing with less influence of tensile and anterior shear forces to achieve wide nerve root decompression and increase surface area of the fusion bed. Another advantage of slip reduction is the correction of the sagittal deformity, which may reduce the incidence of premature adjacent level disc degeneration [29, 30]. On the other hand, powerful slip reduction has been reported to increase risk of neurologic injury [25]. However, successful clinical results have been shown with less than complete reduction: 53% in the present work, 61%–76% in mini-TLIF [24, 31], 50% in ALIF [32, 33], and 75% in the lateral approach [34]. 

Differently from the indirect decompression following circumferential annular release reported by Pan et al. following a mini-TLIF [24], ligamentotaxis following proper disc space distraction has already been described [8, 35, 36] and is considered to be the mechanism of olisthesis reduction and indirect decompression following lateral interbody fusion [8, 36]. In degenerative spondylolisthesis, it is the anterior reconstruction that tends to correct spinal parameters, and the posterior instrumentation functions as a supplementary compression system [20]. But if no restoration of disc height is obtained or great subsidence occurs, and the ligamentous structures have not been put under tension, then supplementation must be added to achieve immediate stability [20]. The addition of an interbody fusion with pedicle instrumentation places the graft in compression and provides maximum immobilization of the motion segment [28, 37]. The cages used in this technique have a wide implant-graft contact area, and interbody grafting immediately reduces motion by an average 70% [28]. Early stabilization following lateral cage insertion provides very low rates of pseudoarthrosis [34] and, in addition to compressive loading, enhances the likelihood of bone ingrowth and success of fusion [38], as has been described following this technique [9, 10]. 

Spondylolisthesis treated with lateral interbody fusion have been reported together with other indications [12, 39, 40] with up to 80% reduction in pain, 75% listhesis improvement, and 98% fusion rate. The common perioperative complication is anterior thigh pain, upper thigh numbness, or hip flexion weakness, which have been reported to be transitory conditions [7, 8, 39, 40]. To mitigate psoas-related and neural complications may be beneficial avoid wanding too much, poor patient positioning, bleeding in psoas, use of monopolar cautery, and preventing wanding too much the retractor inside the psoas muscle, and it is imperative to respect the intraoperative EMG monitoring [41]. Subsidence has been shown not to influence bone fusion rates [6, 42]; however, it may contribute to lack of clinical success [8, 43], at least in the short term. When stenosis is mainly caused by posterior elements (hypertrophic facet joints, calcified ligamentum flavum, or high-grade spondylolisthesis), lateral interbody fusion may be not able to open enough space and relief stenosis symptoms [8]. 

Degenerative spondylolisthesis is one of the most common spine problems and affects mostly the elderly population [44]. Lateral interbody fusion is a minimally invasive option for lumbar spine access which has been reported to be safe and effective, even for elderly patients [45], and for spondylolisthesis specifically, it may be a good alternative for achieving correction of important radiological parameters and clinical improvement with low risk and complication rates.

## Figures and Tables

**Table 1:** Demographic and clinical data.

Patients (*n*)	52
Age (years)	67.6 ± 10.0
Female	38 (73.1%)
BMI (m/kg^2^)	27.4 ± 3.3
Pre-op VAS	77.9 ± 21.8
Pre-op ODI	66.0 ± 16.8
Olisthesis	15.1% ± 5%
Spine levels	52
L1-2	2 (3.8%)
L2-3	9 (17.3%)
L3-4	14 (26.9%)
L4-5	27 (51.9%)
Blood loss (cc)	<50
Surgery duration (min)	73.2 ± 31.4

**Table 2:** Radiological parameters.

	Preoperative	6 weeks	*P* value	12 months	*P* value	24 months	*P* value
Olisthesis	15.1% ± 5.2%	7.4% ± 5.3%	<0.001*	6.7% ± 4.2%	<0.001*	7.1% ± 6.0%	<0.001*
Increase in disc height	—	78% (−29–812)	<0.001*	61% (−21–703)	<0.001*	55% (−28–710)	<0.001*
Segmental lordosis	9.7 ± 3.8°	16.3 ± 5.4°	<0.001*	15.8 ± 6.4°	<0.001*	15.7 ± 7.1°	<0.001*
Global lordosis	42.8 ± 15.0°	48.5 ± 13.8°	0.01*	46.9 ± 12.5°	0.13	46.5 ± 16.2°	0.23
Bone fusion	—	—	—	67.3%	—	86.5%	—

*statistically significant. Values are expressed as mean ± standard deviation or as mean (minimum and maximum).

**Table 3:** Subsidence versus demographic and operative data.

	Subsidence grade		
	0/I	II/III	*P* value
Age (years)	65.5 ± 11.5	71.7 ± 9.4	0.019*
Female	64%	100%	0.041*
L4-5	44.2%	88.9%	0.038*
Olisthesis	15.2 ± 5.5%	14.5 ± 3.6%	0.721

*statistically significant.
